# Mortality associated with third-generation cephalosporin resistance in Enterobacteriaceae bloodstream infections at one South African hospital

**DOI:** 10.1016/j.jgar.2022.03.001

**Published:** 2022-06

**Authors:** Angela Dramowski, Alexander M. Aiken, Andrea M. Rehman, Yolandi Snyman, Sandra Reuter, Hajo Grundmann, J. Anthony G Scott, Marlieke E.A. de Kraker, Andrew Whitelaw

**Affiliations:** a Department of Paediatrics and Child Health, Faculty of Medicine and Health Sciences, Stellenbosch University, Cape Town, South Africa; b Infectious Disease Epidemiology Department, London School of Hygiene and Tropical Medicine; c Department of Medical Microbiology, Faculty of Medicine and Health Sciences, Stellenbosch University, Cape Town, South Africa; d Institute for Infection Prevention and Hospital Epidemiology, Medical Center, University of Freiburg, Freiburg, Germany; e Infection Control Program and WHO Collaborating Center on Patient Safety, University of Geneva Hospitals and Faculty of Medicine, Geneva, Switzerland

**Keywords:** Mortality, Bloodstream infection, Enterobacteriaceae, Extended-spectrum β-lactamase, Antibiotic resistance, South Africa

## Abstract

**Objectives:**

Enterobacteriaceae are common pathogens causing bloodstream infection (BSI) in sub-Saharan Africa and frequently express third-generation cephalosporin (3GC) resistance; however, the impact of 3GC resistance on clinical outcomes is rarely studied.

**Methods:**

We conducted a single-site prospective cohort study at Tygerberg Hospital, Cape Town, South Africa to examine the feasibility of measuring impacts of 3GC resistance in Enterobacteriaceae BSI. We included patients with 3GC-susceptible and 3GC-resistant BSIs and matched each BSI patient to two uninfected patients. We determined the concordance of initial antibiotic treatment with the corresponding isolate's susceptibility profile. We performed exploratory impact analysis using multivariable regression models.

**Results:**

Between 1 June 2017 and 31 January 2018, we matched 177 *Enterobacteriaceae* BSI patients to 347 uninfected patients. Among these BSIs, 35% were phenotypically 3GC resistant. Parameters describing clinical comorbidity showed strong associations with mortality. We found that 18% of 3GC-R and 3% of 3GC-S BSI patient received non-concordant initial therapy. In multivariable Cox regression, we found a mortality impact over their matched patients for both 3GC-R (cause-specific hazard ratio 23.77; 95% CI 5.12–110.3) and 3GC-S (HR 7.49; 95%CI 3.08–18.19) BSI. There was a nonsignificant ratio of these ratios (HR 3.18; 95% CI 0.54–18.70), limited by the small sample size.

**Conclusion:**

This form of impact estimation was feasible in one hospital in South Africa where 3GC-R status was associated with non-concordant initial antibiotic treatment. There was a possible increase in mortality among individuals with 3GC-resistant Enterobacteriaceae, but with broad confidence intervals. These analytical approaches could be applied to larger datasets to improve precision of estimates.

## Highlights

• Global AMR impact estimates require new primary data from African countries.
• We studied impacts of third-generation cephalosporin resistance (3GC-R) in South Africa.
• Uninfected patient matching and clinical outcome measurement was feasible.
• Sequencing demonstrated correlation of phenotypic 3GC-R with ESBL gene presence.
• Estimates of impact size had broad confidence intervals in this single-site study.

## 1 Introduction

Antimicrobial resistance (AMR) is a major global health challenge in the 21st century [1]. A recent major modelling study estimated that there were 1.27 million deaths attributed to AMR in 2019, with sub-Saharan Africa the most intensely affected region globally [2]. Sub-Saharan African countries face many challenges in tackling AMR. First, microbiology facilities and expertise are often limited, so the local ability to recognise and respond to antibiotic-resistant infections is often limited. Second, wider weaknesses in health systems, particularly relating to hospital infection prevention and control and antibiotic stewardship, mean that these countries are highly vulnerable to drug-resistant infections. Third, limited financial resources to access expensive antibiotic treatments means that infections that are treatable in a high-income context are difficult to manage in resource-limited settings.

Bloodstream infections (BSI) caused by Enterobacteriaceae such as *Escherichia coli* and *Klebsiella pneumoniae* are important contributors to neonatal, child and adult mortality in Africa [2], [3], [4], [5], [6]. High rates of third-generation cephalosporin (3GC) resistance are now seen in many African countries [6,7]. This is highly concerning as drugs such as ceftriaxone are widely used across the region, often with limited access to alternative agents. Few estimates exist of the clinical impact of this, or any, form of antibiotic resistance on mortality and length of hospital stay (LOS) in Africa. A recent systematic review on the subject of 3GC-R in sub-Saharan Africa concluded: ‘In Africa, where the prevalence of bacterial sepsis is high … the impact of AMR on patients is predictable, but currently unknown’ [7]. Significant prior AMR impact reports in Africa include work in Senegalese paediatric inpatients [8], Tanzanian children [9] and Ethiopian adults [10]. Our own retrospective pilot study across six African hospitals [11] informed the design of this study. Our study design is modelled on a previous large European study of AMR impacts [12,13].

Methodologically, measuring the impact of AMR is challenging. There is good evidence that antibiotic-resistant infections are more likely to occur in particular patient groups, especially those with prior exposure to antibiotic treatment and/or the healthcare environment, which may in turn be independently associated with poor outcomes—these represent potential confounders. Differences in patient outcomes may also be accounted for by quality of supportive care for critically ill patients and use of antibiotic therapy concordant with the particular organism's antibiotic resistance profile. It is important to consider different comparison groups for AMR impact analyses—either a ‘replacement scenario’ (comparison to susceptible infections) or an ‘addition scenario’ (comparison to no infection) can be used [14]. It is not clear which is the most appropriate comparison group for Enterobacteriaceae [15]. In 2020, the World Health Organization Global Antimicrobial Resistance and Use Surveillance System (WHO-GLASS) group published recommendations on how to go about estimation of the impacts attributable to AMR [16]. These recommendations include: (1) to use bacteraemia infections as a first choice; (2) to examine certain ‘bug-drug’ combinations, in particular extended-spectrum β-lactamase (ESBL)-producing *E. coli* or methicillin-resistant/susceptible *Staphylococcus aureus* (MRSA/MSSA); (3) to examine in-hospital mortality as the primary outcome measure, with 30-day mortality as an alternative; (4) to compare impacts between resistant and susceptible strains of infection with optional use of noninfected patients as an additional comparison group.

In preparation for a multisite study, we conducted a single-site prospective cohort study at one South African hospital to assess the feasibility of gathering linked microbiological, clinical, treatment and outcome data in BSI and matched patients. Contextually, in South Africa at this time the prevalence of 3GC resistance was high in Enterobacteriaceae (25% in *E. coli*, 70% in *K. pneumoniae*), but the prevalence of carbapenem resistance was much lower (8% in *K. pneumoniae*) [17]. We examined whether phenotypic 3GC-susceptibility status correlated with the presence of ESBL genes. We used the resulting data to perform an exploratory impact analysis for 3GC-R status in BSI caused by Enterobacteriaceae applying a parallel-matched cohort design and analytical methods accounting for competing events. Although the number of patients in this preliminary study are modest, this analysis also demonstrates our intended analysis format for a larger follow-on study being conducted across multiple African countries in 2020–2022.

## 2 Methods

### 2.1 Study setting

Tygerberg Hospital is a 1,384-bed public teaching hospital in Cape Town, South Africa (an upper-middle-income country). The facility provides generalist and specialist care to Cape Town's Metro East population and the surrounding district and regional hospitals. Approximately 25% of hospital beds are allocated to neonatal and paediatric care. Annual patient admissions exceed 107 000, with an average occupancy rate of 87% and mean LOS of 6 days.

### 2.2 Study design

All inpatients with laboratory-confirmed mono-microbial *E. coli* or *K. pneumoniae* bloodstream infection (BSI) episodes were prospectively enrolled between 1 June 2017 and 31 January 2018. We chose to focus exclusively on these two species as the most common disease-causing Enterobacteriaceae in this hospital. One research nurse enrolled patients, collected hospital admission date, blood culture date, BSI pathogen, 3GC-susceptibility result, hospital outcome (date of discharge/transfer or in-hospital death) and made follow-up phone calls to determine the 30-day outcome. Recruitment was performed on hospital wards after blood cultures were identified to be positive with a relevant pathogen. Patients were also enrolled if telephonic consent could be obtained from the next of kin (deaths) or patient themselves (after transfer). We classified patients into the following age groups: neonates (0–28 days), infants (29–364 days), children (1–14 years) and adults (>14 years). BSI episodes with blood cultures collected within 48 hours of admission from the community were considered to be community-acquired. Patients with BSI episodes within two days of admission, but with a history of hospitalisation in the preceding 30 days or following transfer in from another facility, were considered to be healthcare-associated BSI. Thereafter, BSI episodes were considered hospital-associated. For each BSI patient, we calculated the Pitt Bacteraemia Score [18], which uses fever, blood pressure, mechanical ventilation requirement, cardiac arrest and mental status to predict risk of mortality from BSI (variables measured from 48 hours prior, up until the day of the positive blood culture). For all patients, we calculated a Charlson Comorbidity Index score [19]. For all patients, a McCabe score [20] was used to classify the underlying illness, as nonfatal, ultimately fatal (expected death in <5 years) or rapidly fatal (expected death in <1 year).

### 2.3 Selection of uninfected matching patients

For each BSI patient identified, two uninfected patients of similar age on the same hospital ward at a similar calendar period were selected. An additional matching constraint was that the uninfected patients’ admissions were required to last at least as long as the time-to-bacteraemia for the corresponding BSI case (interval from hospital admission to blood culture collection). If more than two noninfected patients were eligible for matching, investigators selected those with admission dates closest to that of the BSI patient. For each patient, date of birth, date and ward of admission, date and type of hospital outcome (discharge or in-hospital death) and length of hospital stay were recorded.

### 2.4 Investigation of suspected BSI episodes

At Tygerberg Hospital, blood cultures are obtained from all patients with suspected BSI or severe infection with a focal site (e.g., pneumonia, cellulitis). A single blood culture sample (one bottle) is normally submitted, unless infective endocarditis is suspected. Local guidelines recommend inoculation of 5–10 mL of blood from adults and at least 2 mL of blood from children. Blood cultures are transferred to the on-site National Health Laboratory Service (NHLS) microbiology laboratory for automated processing using the BacT/Alert system (BioMerieux, Marcy l'Etoile, France). If bacterial growth is detected, the sample is subcultured onto appropriate media based on a Gram stain and incubated overnight. Further identification and antimicrobial susceptibility testing of clinically significant isolates is performed with the automated Vitek II system (BioMerieux) or Kirby-Bauer disc diffusion testing if necessary, using annually published CLSI breakpoints [21]. If either cefotaxime or ceftazidime minimum inhibitory concentrations (MIC) were >1 µg/mL, isolates were reported as 3GC-resistant. For purposes of this analysis, isolates classified as ‘intermediate’ 3GC susceptibility status were also considered 3GC-R.

### 2.5 Antibiotic treatment practices

The choice of antibiotic/s for empiric and targeted therapy of BSI in Tygerberg Hospital is at the discretion of attending physicians, although compliance with relevant guidelines is encouraged. For empiric therapy of community-acquired BSI, most local guidelines recommend either a third-generation cephalosporin or a combination of ampicillin + gentamicin. Institutional guidelines on empiric therapy of hospital-acquired BSI recommend piperacillin-tazobactam + amikacin forward–based patients and meropenem (+ vancomycin for suspected central line sepsis or soft tissue infection in hospital) for patients in intensive care units (ICU). We collected data on documented antibiotic use on the calendar date of blood culture performance (day 0) and the subsequent day (day 1), representing the empirical antibiotic therapy used in approximately the first 48-hour period of treatment of bacteraemia, prior to receipt of culture results. One investigator (AA), a practicing clinical microbiologist, compared all the antibiotic medicines documented to have been used against the full susceptibility profile for each individual bacterial isolate. The antibiotic use in this period was summarised as being ‘concordant,’ ‘non-concordant,’ ‘no antibiotics received’ or ‘unable to determine’ for each drug. Use of at least one antibiotic agent with in vitro activity against the relevant bacteria was considered ‘concordant’. Bacterial isolates with intermediate susceptibility to the particular agent used were considered ‘unable to determine’.

### 2.6 Molecular methods

All available isolates were transferred to the University Medical Center Freiburg, Germany for molecular ascertainment of resistance phenotype by whole genome sequencing. Methods used for this work are as described in detail elsewhere [22]. In brief, genomic DNA was extracted using the Roche Pure PCR Template Preparation Kit (Roche Diagnostics, Germany). DNA libraries were prepared using the Nextera XT Library Preparation Kit (Illumina, Germany) and were sequenced with the Illumina MiSeq reagent kit v.2. Sequence reads were assessed for appropriate coverage (>30 × based on mapping to reference genome EC958 [HG941718] for *E. coli* and MGH78578/ATCC700721 [CP000647] for *K. pneumoniae*) and assembly statistics after using SPAdes [23] (N50 >100 000 bp; length ∼4.7–6.2 Mbp; total number of contigs <300). Acquired antimicrobial resistance genes including ESBL genes were detected using ARIBA [24] using the CARD and ResFinder databases. The raw reads have been deposited in the European Nucleotide Archive (ENA; PRJEB46655). We made a final decision on what constituted an ESBL gene according to expert knowledge [25]—we did not consider TEM-1 to represent an ESBL gene.

### 2.7 Statistical analysis

Analyses were conducted using Stata Statistical Software version 16.1 IC (Stata Corp., College station, TX, USA). To understand the feasibility of our study approach, we examined the quality of data at different levels, including the completeness of data for primary outcomes, and hypothesised major confounders. We tested for association between parameters attempting to measure comorbidity and in-hospital survival using log rank test and presented cumulative mortality incidence curves. We used unadjusted Poisson regression with log link to test the association between different categories of use of empirical antibiotic therapy, 3GC-resistance status and mortality by generating a relative risk [26].

In exploratory analyses, we used principles previously used in our retrospective pilot study [11] and elsewhere [12]. For the analysis of AMR impact, the primary exposure was phenotypic 3GC resistance status (resistant or susceptible) and outcomes of interest were mortality (hospital mortality and 30-day mortality). The overall impact of resistant BSI or susceptible BSI on our outcomes was evaluated by contrasting them against their uninfected matches in two separate regression models (described below for each outcome). These estimates were used to generate a ‘resistant vs. susceptible’ ratio of ratios to determine the impact of 3GC resistance [27]. Robust standard errors were used in all models to account for the matched design. The low number of events for pathogen-specific BSIs precluded species-specific subgroup analyses.

Cause-specific (hospital mortality and hospital discharge) Cox regression models were used to estimate hazard ratios (HR) to explore the effect of 3GC resistance on time to hospital outcome [28]. The proportional hazards assumption of the Cox models was assessed using Schoenfeld residuals. BSI cases’ follow-up time commenced on the date of blood culture. Matched patients’ follow-up time commenced on the equivalent day of their admission to their BSI case, to prevent immortal time bias. We also used Fine and Gray's extended Cox regression model to simultaneously consider death and discharge from hospital as two possible competing events and generated the subdistribution HR for death [29]. We used generalised linear models with Poisson distribution and log link to estimate the relative risk of 30-day mortality [26]. Our multivariable models included all available variables representing the pre-existing health state of individual patients (age category, TB treatment, HIV status, McCabe score and categorical Charlson Comorbidity Index) to account for potential confounding. We did not include variables representing the state of acute illness at time of assessment (Pitt bacteraemia score), as these were considered to lie on the causal pathway between infection and mortality. We did not include parameters representing bacterial species or source of infection in the main adjusted analysis as non-bacteraemic matching patients had no corresponding data. Under a missing at random assumption, to include 81/524 (15%) patients with unknown HIV status, we used multiple imputation with chained equations to impute categorical HIV status in 10 imputation datasets. Imputation models included hospital outcome, species, BSI status, age category, TB status, McCabe score and Charlson comorbidity index.

## 3 Results

### 3.1 Study subjects (Table 1)

We recruited 177/190 of the eligible *E. coli* and *K. pneumoniae* bacteraemia patients in this hospital over the study period, for an overall recruitment success of 93%. Reasons for noninclusion of potentially eligible patients included death before enrolment (n=6), transfer to another hospital before enrolment (n=6) and declined consent (n=1). There were no repeat positive cultures within the same patient. For *E. coli* BSI, 25 out of 106 (24%) and, for *K. pneumoniae* BSI, 37 out of 71 (52%) were classified as 3GC-resistant; this included one *E. coli* isolate with intermediate 3GC susceptibility status. Two of the 177 isolates had reduced carbapenem susceptibility—one *K. pneumoniae* isolate (meropenem MIC of 2 μg/mL; intermediate) and one *E. coli* isolate (meropenem MIC of 4 μg/mL; resistant). All other isolates of both species were susceptible to meropenem and imipenem (MIC ≤1 μg/mL).

Across both species combined, fewer community-acquired BSI were caused by 3GC-R organisms (18%, 6/33) than healthcare-associated (35%, 15/43) or hospital-acquired (41%, 41/101) infections. Each BSI patient was matched to two uninfected ward patients, though eight of the matched patients were subsequently excluded based on not meeting one or more matching criteria. This represented a total of 177 Enterobacteriaceae BSI (35% 3GC resistant) and 346 uninfected matched patients (see Table 1). Adults were the predominant age group, with neonates and children making up approximately 30% of study subjects and 53% of BSI patients were female. Among all BSI patients, the presumed sources of infection were most commonly intra-abdominal (67/177; 38%), pneumonia (37/177; 21%) and urinary tract infections (36/177; 20%). Amongst BSI patients with a known HIV status, 23/126 (18%) were known to be HIV-infected, with the majority of these (19/23; 83%) currently receiving antiretroviral therapy (ART).

**Table 1:** Profile of BSI patients and matching patients

	3GC-S cohort		3GC-R cohort	
	3GC-S BSI(*n* = 115)	Matched uninfected patients(*n* = 227)	3GC-R BSI(*n* = 62)	Matched uninfected patients(*n* = 120)
**Individual characteristics**				
Sex, male (*n*, %)	51 (44%)	110 (48%)	32 (52%)	55 (46%)
Age group (n, %)				
Neonates (0-28 d)	14 (12%)	29 (13%)	9 (15%)	25 (21%)
Infants (29-364 d)	6 (5%)	16 (7%)	9 (15%)	12 (10%)
Children (1-14 y)	5 (4%)	9 (4%)	5 (8%)	11 (9%)
Adults (>14 y)	90 (78%)	173 (76%)	39 (63%)	72 (60%)
HIV status (n, %)				
Negative	65 (57%)	181 (80%)	38 (61%)	98 (82%)
Positive, on ART	10 (9%)	18 (8%)	9 (15%)	11 (9%)
Positive, not on ART	2 (2%)	2 (1%)	2 (3%)	4 (3%)
Unknown (inc. exposed children)	38 (33%)	26 (11%)	13 (21%)	7 (6%)
On TB treatment (any)	3 (3%)	10 (4%)	3 (5%)	3 (3%)
McCabe score				
Non-fatal	92 (80%)	203 (89%)	45 (73%)	104 (87%)
Ultimately fatal	12 (10%)	24 (11%)	8 (13%)	15 (12%)
Rapidly fatal	11 (10%)	0 (0%)	9 (15%)	1 (1%)
Charlson Comorbidity Index score: Median (IQR)	1 (0–2)	0 (0–2)	2 (0–3)	0 (0–2)
				
**Characteristics of BSI**				
Bacterial species				
* E. coli*	81 (70%)	N/A	25 (40%)	N/A
* K. pneumoniae*^a^	34 (30%)		37 (60%)	
Community-acquired	27 (23%)	N/A	6 (10%)	N/A
Healthcare associated	28 (24%)		15 (24%)	
Hospital-acquired	60 (52%)		41 (66%)	
Admission to infection, days: median (IQR)	2 (0–10)	N/A	7 (0–14)	N/A
Presumed BSI source (n, %)				
Intra-abdominal infection	51 (44%)	N/A	16 (26%)	N/A
Genito-urinary infection	22 (19%)		14 (23%)	
Pneumonia	23 (20%)		14 (23%)	
Other infection site^b^	8 (7%)		10 (16%)	
No focus identified	11 (10%)		8 (13%)	
Pitt bacteraemia score: median (IQR)	2 (0-5)	N/A	3 (1-6)	N/A
Outcomes				
Hospital outcome				
Discharged/transferred out	86 (75%)	221 (97%)	39 (63%)	118 (98%)
Died	29 (25%)	6 (3%)	23 (37%)	2 (2%)
Length of stay in days: median (IQR)	9 (4–23)	8 (6–17)	10.5 (6–18)	12.5 (6–20)
30-d outcome				
Alive	82 (71%)	214 (94%)	35 (56%)	113 (94%)
Died	33 (29%)	10 (4%)	27 (44%)	4 (3%)
Unknown	0	3 (1%)	0	3 (3%)

ART, antiretroviral therapy; BSI, bloodstream infection; ICU, intensive care unit; IQR, interquartile range.

a *K. pneumoniae* includes four isolates subsequently identified as *K. variicola* by WGS.

b Other infection = skin and soft tissue, meningitis, dysentery/diarrhoea.

### 3.2 Data completeness

**Fig. 1:**
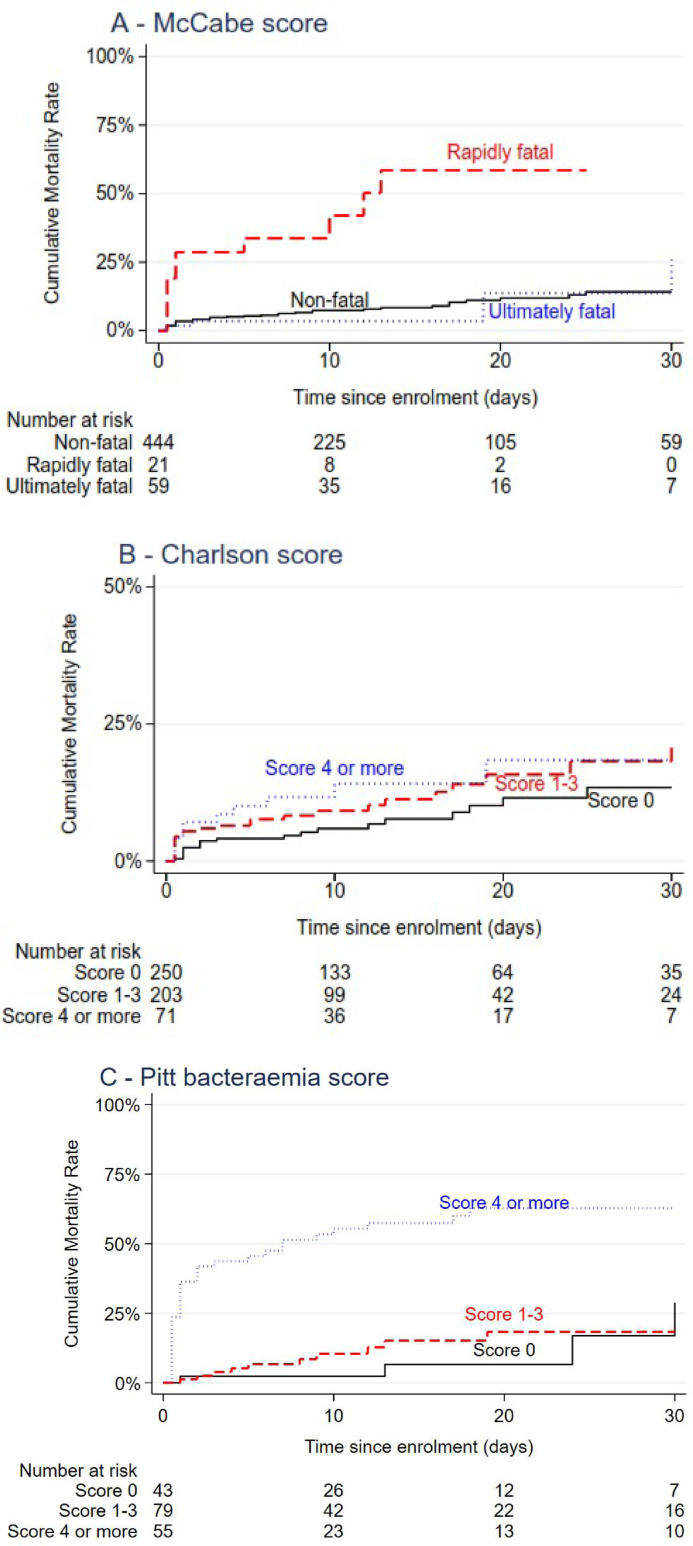
(A and B) Relationship of McCabe score and Charlson Comorbidity Index with in-hospital mortality, all participants. (C) Relationship of Pitt Bacteraemia score with mortality, bacteraemic participants only.

There was a high level of completeness for the clinical and outcome parameters of interest for this study. These included data for the main outcomes of in-hospital mortality (100% complete) and 30-day mortality (99% complete) and also for parameters describing clinical comorbidity (Charlson Comorbidity Index and McCabe score) and acute illness (Pitt bacteraemia score)—all were 100% complete. Across the whole cohort of BSI and noninfected matching patients, for both the McCabe score and the Charlson Comorbidity Index, these parameters showed an association with the risk of in-hospital mortality (log rank test for equality of survival curves, *P* values are <0.0001 for McCabe scores and *P* = 0.0158 for Charlson Comorbidity Index; see Fig. 1A and 1B). For BSI patients only, the Pitt bacteraemia score was also associated with risk of in-hospital mortality (log rank test for equality of survival curves, *P* <0.0001,; see Fig. 1C)

### 3.3 Empirical antibiotic therapy (Tables 2 and 3)

Antibiotic treatment regimens used in the initial (day 0 and day 1) treatment period are shown in Table 2. Use of carbapenem-containing empirical regimens was common (37%, 66/177 BSI patients), as was use of a regimen of piperacillin-tazobactam combined with amikacin (20%, 36/177 BSI patients). Use of empirical regimes containing a cephalosporin antibiotic was limited (15%, 26/177). A small number of patients did not receive any antibiotic treatment in this initial period (6.2%, 12/177)—these patients had high risk of mortality (83%, 10/12). In terms of the microbiological concordance of treatments used, the large majority (144/177, 81.4%) of bacteraemic patients received antibiotic treatment that was microbiologically concordant with the specific isolate identified (Table 3). Only a small proportion of bacteraemia patients received antibiotic treatment that was microbiologically non-concordant (16/177, 9.0%). Receipt of non-concordant antibiotic treatment in this period was associated with 3GC-resistance status (RR 2.58, 95% CI 1.72–3.85, *P* = 0.001). However, there was no evidence of a mortality impact associated with non-concordant empirical antibiotic treatment (mortality with non-concordant initial treatment 1/15 [6.7%], mortality with concordant initial treatment 39/144 [27.1%], RR 0.25, 95% CI 0.04–1.67).

**Table 2:** Empirical antibiotic regimes used (day 0 + day 1) for 177 BSI patients

	Hierarchically listed agents	Concordant	Non-concordant	Unable to determine	Total
Carbapenem					
	Meropenem	16			16
	Meropenem + other agent(s)	18	1		19
	Imipenem	10			10
	Imipenem + other agent(s)	7			7
	Ertapenem	10			10
	Ertapenem + other agent(s)	4			4
β-lactam/β-lactam inhibitor (BLBI)					
	Piperacillin-tazobactam	2	1		3
	Piperacillin-tazobactam + amikacin	33		3	36
	Piperacillin-tazobactam + other agent(s)	5			5
	Co-amoxiclav	14	5	3	5
	Co-amoxiclav + other agent(s)	1	1		2
Third-generation cephalosporins					
	Ceftriaxone	10	4		14
	Ceftriaxone + other agent(s)	8			8
	Other regimes with 3GC	2	2		4
Other penicillins					
	Ampicillin + gentamicin +/- other agent(s)	2	1		3
Quinolones					
	Ciprofloxacin +/- other agent(s)	2			2
					
No antibiotic use recorded on day 0 or day 1					12
	Total (% of total)	144 (81%)	15 (8.5%)	6 (3.4%)	177

NOTE: Regimes are displayed hierarchically based on principal treatment agent used, with agents in descending order (as per table sequence) such that each patient is represented once only. For example, if a patient received a regime containing both ‘meropenem’ and ‘ceftriaxone’, they are listed under ‘meropenem + other agent(s)’ only.

3GC, third-generation cephalosporin; BSI, bloodstream infection.

**Table 3:** Concordancy of initial antibiotic therapy (day 0–day 1)

	3GC-S	3GC-R	Total	Relative risk
Received concordant initial antibiotic treatment	103	41	144 (81.4%)	Baseline
Received non-concordant initial antibiotic treatment	4	11	15 (8.5%)	2.58 (1.72–3.85)
Did not receive any antibiotic in the first 48 h	5	7	12 (6.8%)	2.05 (1.19–3.53)
Unable to determine antibiotic concordancy, including intermediate drug susceptibility status	3	3	6 (3.4%)	Not tested
Total	N = 115	N = 62	N = 177	

3GC-R, third-generation cephalosporin-resistant; 3GC-S, third-generation cephalosporin-susceptible; BSI, bloodstream infection.

**Table 4:** Phenotypic 3GC status vs. presence of ESBL genes

		Whole genome sequencing result			
Phenotypic 3GC susceptibility result		ESBL genenot detected	Any ESBL gene detected	WGS not performed	Total

	3GC-S	74 *E. coli*	2 *E. coli*	5 *E. coli*	*E. coli* = 81
			- 1 CTX-M-15		
			- 1 CARB-2		
		26 *K. pneumoniae*	1 *K. pneumoniae*	7 *K. pneumoniae*	*K. pneumoniae* = 34
			- 1 SHV-27		All = 115

	3GC-R		19 *E. coli*	1 *E. coli*	*E. coli* = 25
		5 *E. coli*^a^	- 11 CTX-M-15		
			- 5 CTX-M-14		
		2 *K. pneumoniae*	- 3 CTX-M-27		
			32 *K. pneumoniae*	3 *K. pneumoniae*	*K. pneumoniae* = 37
			- 30 CTX-M-15		
			- 1 CTX-M-15 and OXA-10		
		- 1 CTX-M-3		All = 62

	*E. coli* = 80	*E. coli* = 20	*E. coli* = 6	*E. coli* = 106
	Total	*K. pneumoniae* = 28	*K. pneumoniae* = 33	*K. pneumoniae* = 10	*K. pneumoniae* = 71
		All = 108	All = 53	All = 16	All = 177

NOTE: *K. pneumoniae* includes six isolates subsequently identified as *K. variicola* by WGS.

ESBL, extended-spectrum β-lactamase.

a Includes one *E. coli* isolate with intermediate 3GC susceptibility status.

**Table 5:** Impact of third-generation cephalosporin resistance on mortality amongst all Enterobacteriaceae BSI, adjusted analyses

	In-hospital outcomes cause-specific HR (95% CI)		In-hospital outcomes subdistribution HR (95%CI)		
Comparison	Cox model (death)	Cox model (discharge)	Fine + Gray model (death)	Fine + Gray model (discharge)	30-day mortality RR (95% CI)
3GC-R BSI vs. matched patients	23.77(5.12–110.29)	0.59(0.41–0.86)	29.51(6.51–133.8)	0.34(0.23–0.51)	11.75(4.23–32.66)
3GC-S BSI vs. matched patients	7.49(3.08, 18.19)	0.60(0.47, 0.78)	9.78(4.00–23.90)	0.43(0.33, 0.56)	5.58(2.80–11.11)
3GC-R BSI vs. 3GC-S BSI	3.18(0.54, 18.70)	0.98(0.63, 1.54)	3.02(0.52–17.46)	0.79(0.49, 1.28)	2.11(0.61–7.22)

NOTE: All models are adjusted for age category, TB treatment, HIV status (including multiple imputation where HIV status unknown), McCabe score and categorical Charlson Comorbidity Index.

3GC-R, third-generation cephalosporin-resistant; 3GC-S, third-generation cephalosporin-susceptible; BSI, bloodstream infection; CI, confidence interval; HR, hazard ratio.

### 3.4 Molecular detection of ESBL genes (Table 4)

Out of 177 Enterobacteriaceae BSI cases, whole genome sequencing (WGS) was performed on 161 (91%) of isolates. All bacterial isolates had their phenotypic identification confirmed to at least the genus level. Six putative *K. pneumoniae* isolates were identified as *K. variicola*, a closely related species indistinguishable by routine phenotypic laboratory testing—we therefore retained these isolates with the *K. pneumoniae* isolates in further analysis. The *E. coli* collection was dominated by the globally distributed ST131 Complex (23/100 isolates) and ST69 Complex (16/100 isolates), whereas there were no particular dominant clones in *K. pneumoniae*. In phenotypically 3GC-R isolates, we identified ESBL-encoding genes in 19/24 *E. coli* and 32/34 *K. pneumoniae* isolates sequenced. Overall, 88% (51/58) of 3GC-R isolates had ≥1 detectable ESBL-encoding gene. CTX-M elements were the most common genetic basis for ESBL production in both species, with CTX-M-15 being the most common of these. In isolates phenotypically identified as 3GC-S, we identified ESBL-encoding elements in only 2/76 *E. coli* (CTX-M-15 and CARB-2 genes each detected in one isolate) and 1/27 *K. pneumoniae* (SHV-27 gene detected). As we were uncertain about the functional status of these genes, we retained the phenotypic classification for these isolates in other analyses.

### 3.5 Exploratory analysis of AMR impacts (Figure 2 and Table 5)

**Fig. 2:**
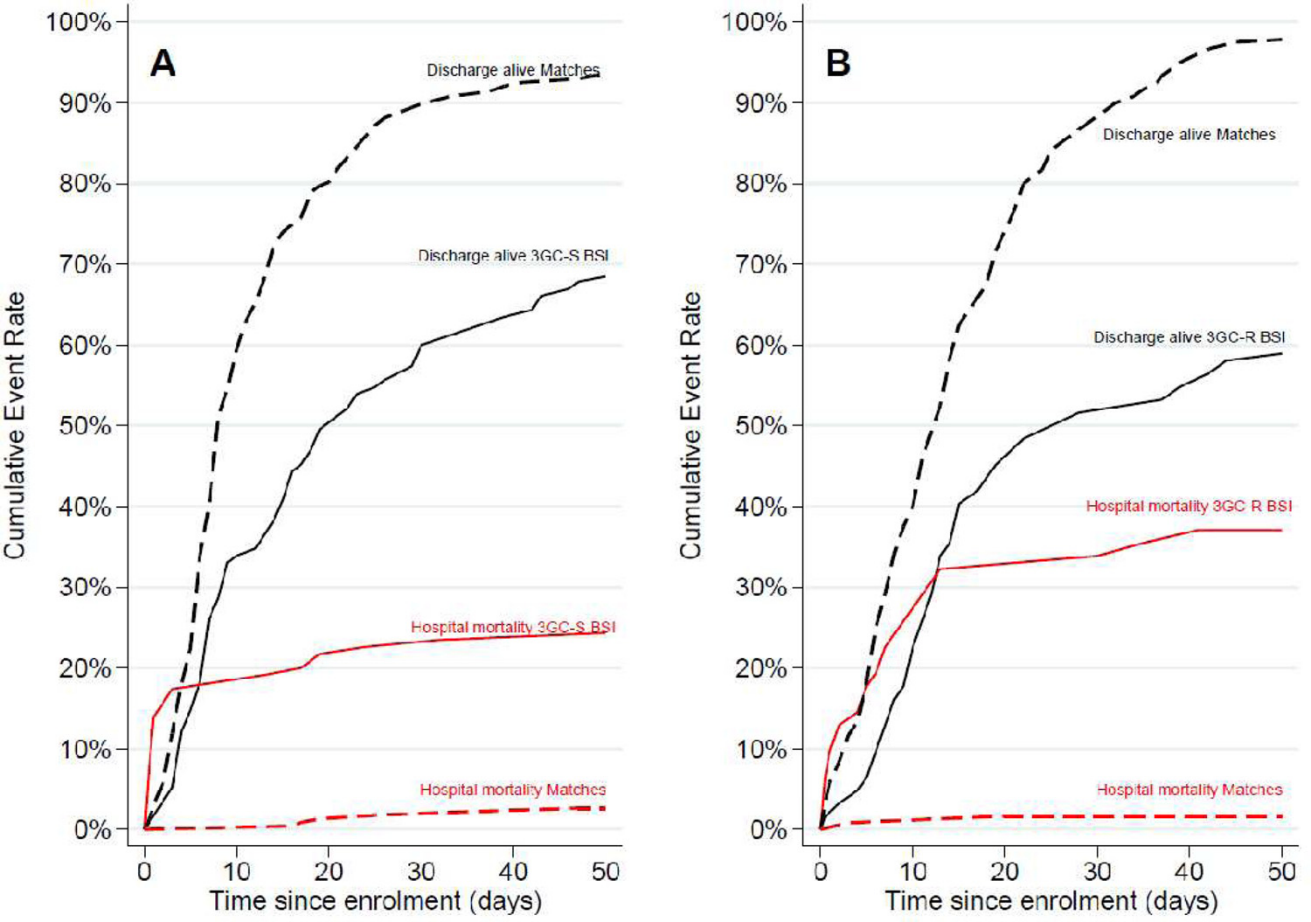
Unadjusted cumulative mortality incidence of hospital outcomes for BSI and noninfected patients. (A) Third-generation cephalosporin-susceptible BSI and matching patients. (B) Third-generation cephalosporin-resistant BSI and patients.

We found that 3GC-R BSI had a substantially increased hazard of in-hospital mortality over their matched uninfected patients (cause-specific hazard ratio 23.77; 95% CI 5.12–110.29, test for the proportional hazards assumption χ^2^ > 0.16 for each imputation dataset). This impact was strengthened by an association of 3GC-R BSI with reduced rate of hospital discharge (csHR 0.59; 95% CI 0.41–0.86, test for the proportional hazards assumption χ^2^ > 0.84), meaning that these BSI patients had prolonged admissions. There was also an increased hazard of in-hospital mortality for patients with 3GC-S BSI over their matched uninfected patients (csHR 7.49; 95% CI 3.08–18.19, test for the proportional hazards assumption χ^2^ < 0.02), though this was lower than for the resistant cohort, with a similar reduced rate of hospital discharge (test for the proportional hazards assumption χ^2^ > 0.98). See Fig. 2A and 2B. The overall ratio of csHRs for inpatient mortality in 3GC-R vs. 3GC-S BSI was 3.18 (95% CI 0.54–18.70), and for hospital discharge it was 0.98 (95% CI 0.63–1.54). Although the point estimate indicates a possible mortality impact of 3GC-R over 3GC-S, the wide confidence intervals crossing the value of 1.0 means that these data do not give evidence of a direct association of 3GC-R with mortality risk in this setting. The ratio of csHRs for discharge was close to 1.0, indicating no difference in LOS by resistance status.

In Fine and Gray models for inpatient death, the subdistribution HRs for both resistant and susceptible forms of infection were higher than the corresponding event-specific Cox models for mortality. The ratio of subdistribution HRs also showed a raised point estimate with broad confidence intervals crossing the unity value (sdHR 3.02, 95% CI 0.52–17.46).

Similar to the findings for in-hospital mortality, we found that after adjustment, both 3GC-R and 3GC-S Enterobacteriaceae BSI had substantially increased 30-day mortality risk over their matched uninfected patients. Although the relative risk for 3GC-R (RR 11.75, 95% CI 4.23–32.66) was higher than the corresponding risk for 3GC-S (RR 5.58, 95% CI 2.80–11.11), the ratio of these risks was again not significantly different (ratio of RR 2.11, 95% CI 0.62–7.22).

## 4 Discussion

We followed a methodological approach for AMR impact measurement previously employed in a large European study [12] and the recommendations made by the WHO-GLASS group [16]. At one large hospital in South Africa, we prospectively enrolled patients with bloodstream infection and matched noninfected patients, gathering relevant clinical and outcome data. The central finding of this study was that our approach was feasible to conduct, with plausible preliminary information being generated along several key dimensions. We found good evidence that parameters designed to measure comorbidity were associated with mortality risk. In this collection of bacterial isolates, phenotypic 3GC-resistance status correlated well with the presence of ESBL-encoding genes. The profile of ESBL genes identified fits with previously descriptions in African countries [30].

We performed exploratory impact analyses to demonstrate our planned methodological approaches for a larger future analysis. The central point estimate for mortality impact in this study (HR 3.18 for hospital mortality outcome) suggests there could be a substantial mortality impact associated with 3GC resistance. This estimate has broad confidence intervals (95% CI 0.54–18.70) due both to the study design and the modest sample size. Using a ratio-of-ratios as the main impact estimate combines the uncertainty from both parameters, leading to a wide confidence interval. We also show estimates using an alternative Fine and Gray model that accounts for the competing risk from alternative censoring events. As bacteraemia infections lead to prolonged admissions amongst survivors, bacteraemia patients typically spend a longer period of time in hospital than the noninfected matched patients, so in a Fine and Gray model for death, bacteraemia patients experience a higher per day risk of death than seen in a corresponding Cox model. These are alternative models for presenting these data, and they give similar qualitative conclusions.

This preliminary study does not, in itself, indicate a robust mortality impact of this form of antibiotic resistance or non-concordant initial antibiotic treatment in this hospital. We note that there were few bacteraemia patients who received microbiologically non-concordant treatment in this hospital (only 9% of total), and this was associated with 3GC-resistance status in the bacterial isolates. As third-generation cephalosporin antibiotics were relatively rarely used (only 15% in initial 48-hour treatment period) and few patients received non-concordant initial therapy, this means that the scope to detect a true impact of 3GC-R or non-concordant therapy was limited in this hospital in South Africa. We anticipate that in many hospitals elsewhere in sub-Saharan Africa, larger proportions of patients with 3GC-R BSI caused by Enterobacteriaceae are likely to receive a cephalosporin antibiotic as part of non-concordant initial antibiotic therapy, such that an association between 3GC resistance and a poor clinical outcome could plausibly be more pronounced elsewhere in the continent than seen here.

This study has important strengths that are mirrored in our ongoing main multisite study. These include a prospective methodology, largely following WHO-GLASS best-practice recommendations; collection of contemporaneous clinical data to attempt to control for important confounders; and gathering information on actual per-patient antibiotic usage to allow us to observe effects of actual treatment practices. We performed molecular confirmation of bacterial speciation and the presence of ESBL genes, giving us confidence that 3GC-R phenotype is largely synonymous with the presences of ESBL genes. The main limitation of this study is its modest sample size, reflecting the single-hospital prospective format. The Cox model for mortality comparing 3GC-S vs. matches indicated evidence of violation against the proportional-hazards assumption, because of bunching of event times among the matched cohort. However, sensitivity analysis using Poisson regression with Lexis expansion of follow-up time revealed almost identical hazard ratios indicating the Cox estimates should not automatically be assumed biased, although they might be interpreted cautiously.

Most studies of AMR impact compare resistant and susceptible forms of infection directly without use of noninfected comparison patients. However, such approaches only provide the lower limit of the possible impact of AMR through implicit adoption of the replacement scenario, where it is assumed that reduction of AMR will not decrease the overall number of infections but will only decrease resistance proportions among these infections. This approach ignores the addition scenario, where it is assumed that reduction in AMR results in reducing the number of drug-resistant infections, while the number of drug-susceptible infections remains stable. At present it is unclear which of these scenarios is more appropriate for drug-resistant Enterobacteriaceae [15]. In the most general terms, the impact of community-acquired resistant infections may be best considered under the replacement scenario, whilst hospital-acquired infections are often best considered under the addition scenario—our study represents a mixture of these groups. The WHO-GLASS recommendations propose inclusion of matched noninfected patients in these types of analysis, as we have done.

Prospective evaluations of the impacts of AMR in bacteraemia patients have rarely been conducted in African countries, but this study shows that it can be achieved with relatively modest data collection requirements. A recent major modelling study commented on the severe scarcity of data linking laboratory outcomes to clinical outcomes such as death [2]. We achieved good recruitment and excellent data completeness for Enterobacteriaceae BSI patients in one South African hospital. We confirmed the presence of ESBL genes in the majority of 3GC-R isolates, and exploratory analysis indicated that there might be a substantial mortality impact associated with 3GC-R status. A full-scale multisite study following this same methodology is now in progress in eight African hospitals, which we hope will provide a more precise assessment of this form of AMR impact.

## Appendix Supplementary materials

Image, 1
